# Potential Application of Cord Blood-Derived Stromal Cells in Cellular Therapy and Regenerative Medicine

**DOI:** 10.1155/2012/365182

**Published:** 2012-11-14

**Authors:** Simone Maria Kluth, Teja Falk Radke, Gesine Kogler

**Affiliations:** Institute for Transplantation Diagnostics and Cell Therapeutics, Heinrich Heine University Medical Center, 40225 Duesseldorf, Germany

## Abstract

Neonatal stromal cells from umbilical cord blood (CB) are promising alternatives to bone marrow- (BM-) derived multipotent stromal cells (MSCs). In comparison to BM-MSC, the less mature CB-derived stromal cells have been described as a cell population with higher differentiation and proliferation potential that might be of potential interest for clinical application in regenerative medicine. Recently, it has become clear that cord blood contains different stromal cell populations, and as of today, a clear distinction between unrestricted somatic stromal cells (USSCs) and CB-MSC has been established. This classification is based on the expression of DLK-1, HOX, and CD146, as well as functional examination of the adipogenic differentiation potential and the capacity to support haematopoiesis *in vitro* and *in vivo*. However, a marker enabling a prospective isolation of the rare cell populations directly out of cord blood is yet to be found. Further analysis may help to reveal even more subpopulations with different properties, which could be useful for the directed application of these cells in preclinical models.

## 1. Multipotent Stromal Cells (MSCs)

In 1966, Friedenstein et al. described for the first time a population of fibroblastoid, bone marrow-derived cells with stem cell characteristics and a multipotential differentiation capacity [1]. These nonhaematopoietic, spindle-shaped cells, originally referred to as “colony forming unit fibroblasts” (CFU-Fs), had the potential for ectopic bone formation and differentiation towards the adipogenic and chondrogenic lineage [2].

In accordance with the observed differentiation potential, Caplan et al. proposed the term “mesenchymal stem cells” and the abbreviation MSC in 1991. Due to controversial discussion of the “true” mesenchymal or stem cell properties, MSC later has also been used as abbreviation for “mesenchymal stromal cell” or “multipotent stromal cell”. As of today, MSCs are still discussed as being true stem cells [3], mesenchymal stromal cells [4], multipotent skeletal progenitors [5], or related to pericytes [6].

Although a common progenitor has not yet been found, the expression of Stro-1, CD271, and CD146 is controversially discussed as potential candidates in aspect of prospective isolation [7–11]. Nevertheless, MSCs were successfully isolated from various tissues, such as bone marrow [1], cord blood [12], and adipose tissue [13]. There was no clear distinction between the different cell types, neither regarding their *in vitro-* nor their *in vivo*-differentiation potential. All of these cells were able to expand and differentiate *in vitro*, but expansion capacity in cell culture was shown to be limited and finally resulted in growth arrest, therefore questioning the classification as “stem cell.” In 2006, the International Society for Cellular Therapy (ISCT) addressed this difficulty [4], and the term “mesenchymal stem cells” was changed to “mesenchymal stromal cells,” which better reflects the cells properties. Since then, MSCs are defined by their morphology (fibroblastoid shape, plastic adherence, and growth as monolayer), differentiation capacity* in vitro* (into adipogenic, osteoblastic, and chondrogenic lineage), and expression of surface antigens (positive expression of CD105, CD73, and CD90, while lacking expression of CD34, CD45, CD14, CD11b, CD79, CD19, and HLA-DR). It has to be noted that this immunophenotype is not exclusive to MSC (it is also expressed on fibroblasts) but rather excludes haematopoietic stem cells (CD34^+^), leukocytes (CD45^+^, CD14^+^, CD11b^+^, CD79^+^, and CD19^+^), and endothelial cells (CD31^+^).

*In vivo*, MSCs represent a rare population with multiple, partially still unknown, functions, for example, as a part of the haematopoietic niche in the bone or in support of repair mechanisms after injury. 

## 2. Cord Blood

In fetal development, the unborn child is provided with nutrients and oxygen via the placenta, but contrary to popular beliefs, mother and child are not directly connected by the umbilical cord. Though the umbilical cord vein transports oxygen and nutrient-rich blood to the child, the umbilical cord artery carries by-products and carbon dioxide back to the placenta. Thus, blood cells cannot pass the placenta-uterus border, which represents a semipermeable frontier between maternal and fetal circulation. In particular, haematopoietic stem cells (HSCs) circulate during development and are enriched in fetal blood in comparison to adult peripheral blood. These immature HSCs have been used in treatment of haematological diseases as alternative to bone marrow-derived HSCs since the first successful transplantation in 1988 by Gluckman et al. [14].

Briefly, the cord blood (CB) is gathered from the umbilical cord after cord clamping by sterile puncture of the umbilical vein and transferred into special transport bags. After processing (volume reduction, HLA-typing, and sterility control), the transplant is stored in liquid nitrogen for further use. In comparison to bone marrow, cord blood is more quickly available, and it can be collected without any risk or pain for mother and child. Due to its immaturity, it can be transplanted with up to 2 HLA mismatches between transplant and patient without higher risk of *graft versus host disease* (GvHD).

However, besides these haematopoietic stem cells, CB also contains various other cell types that might be of potential interest with regard to regenerative medicine or tissue engineering, including cells with MSC-like properties.

## 3. Cord Blood-Derived Stromal Cells

The occurrence of nonhaematopoietic, multipotent stromal cells in cord blood was first described by Erices et al. in 2000 [12]. In 2004, Kögler et al. published a protocol for the generation of stromal cells from CB. Basically, the fraction of the mononucleated cells is isolated using a Ficoll gradient centrifugation followed by red blood cell lysis and subsequent cultivation in culture flasks with serum-rich media. In approximately 40%–45% of the processed CBs, formation of colonies (1–11 per CB) of adherent cells with MSC-like morphology could be detected within 7–21 days [15]. 

In comparison to bone marrow-derived stromal cells (BM-MSCs), these cells, originally termed USSCs (unrestricted somatic stromal cells), were characterized by a higher differentiation potential as well as higher proliferative potential and longer telomeres [15]. Regarding the immunophenotype, no significant differences were observed between stromal cells from cord blood or bone marrow. Both cell types showed the “MSC phenotype” as defined by the ISCT (see earlier), though CB-derived stromal cells showed a higher support of HSC in cocultures *in vitro*. 

While initially it was discussed whether or not these cells might be of embryonic origin, it was later shown that they neither express the POU5F1-gene OCT4a [16] (which is one of the most important embryonic stem cell markers) nor Nanog, SOX2, or hTERT [17] which are abundantly expressed in pluripotent stem cells. In addition, these cord blood-derived stromal cells did not show an intrinsic tumorigenicity after subcutaneous injection in immune-deficient nude mice [18]. 

## 4. Cord Blood Contains Different Stromal Cell Populations

Though described as a single-cell population with a high osteogenic, chondrogenic, as well as adipogenic differentiation potential in 2004, later experiments revealed that cord blood-derived stromal cell lines might have different properties. In 2010, our group was able show that cord blood contains at least two different stromal cell populations which were classified as USSC and CB-MSC. Both populations are isolated by the protocol described earlier; however, to date a prospective isolation of either USSC or CB MSC has not been possible. 

The classification of these two cell types requires examination of the differentiation potential as well as the gene expression profile [17]. As of now, expression profiles of DLK-1, HOX-Genes, and CD146, as well as functional differences regarding adipogenic differentiation and support of haematopoietic cells have been established as discriminating markers.

In contrast to CB-MSC and BM-MSC, USSCs lack the adipogenic differentiation potential *in vitro *and exhibit a higher expression of DLK-1. DLK-1 was first described as adipogenic inhibitory factor in a mouse model [19]. It belongs to the Delta Notch family, lacking the characteristic DSL motif which is necessary for interaction with the NOTCH receptors [20]. During embryogenesis, DLK-1 is highly expressed [21, 22] and seems to be important for skeletal development [23]. After birth, it is downregulated in most tissues of the body, except some endocrine tissues [24]. Different publications could show that the overexpression of DLK-1 blocks the adipogenic differentiation potential of human MSC, as well as osteogenic and chondrogenic differentiation to a certain point [25–27]. Up till now, the role of DLK-1 in USSC is not fully understood and needs to be further defined. 

Aside from discriminating USSC and CB-MSC by their ability to differentiate into adipogenic cells and the inverse correlation of DLK-1 expression, the expression of HOX (homeobox containing) genes provided a further possibility to distinguish between USSC and CB-MSC [28]. While CB-MSCs, similar to BM-MSCs, are characterized by the expression of various HOX genes, USSCs display a negative HOX expression profile. HOX genes encode for transcription factors that determine the positional identity along the body axis during embryogenesis and are characterized by the homeodomain [29]. In human, 39 different HOX genes that are divided into 4 HOX clusters (HOXA, HOXB, HOXC, and HOXD) are known. Leucht et al. could show that the specific HOX gene pattern of a cell (HOX code) might be important for transplantation issues [30]. They demonstrated that the transplantation of a HOX negative cell into a HOX positive tissue leads to a HOX-code adaption. HOX positive cells, by contrast, are not able to downregulate the expression after exposure to a HOX-negative environment. 

After examining a high number of different CB-derived cell lines, the expression of CD146 turned out to be a further discrimination factor between USSC and CB-MSC [31]. CD146, also known as melanoma-associated cell adhesion molecule (MCAM), is expressed on pericytes [6] and stromal cells from bone marrow with osteogenic differentiation potential *in vivo* [11]. While CB-MSCs, as well as BM-MSCs, were characterized by a strong expression of extracellular CD146, the expression on USSC was comparatively lower in flow cytometric analysis. 

Finally, apart from adipogenic differentiation, USSC showed another functional difference to CB-MSC or BM-MSC. While MSCs in general possess the ability to support haematopoiesis, USSCs lead to a higher expansion rate of CD34^+^-selected haematopoietic stem cells in *in vitro *cocultures, presumably due to a large variety of secreted cytokines [32].

In cooperation with the German Cancer Research Centre (DKFZ), it was demonstrated that coinjection of USSC together with CD34^+^ cells in a NOD/SCID mice model enhanced the engraftment of the human haematopoietic cells to the bone marrow as compared to CD34^+^ cells alone [18], while the stromal cells themselves did not engraft into the host.

These results clearly show that USSC and CB-MSC are two distinct cell populations with different properties that need to be further defined for potential clinical application of these cells.

## 5. Clonal Populations

Various publications deal with the problem that MSC populations display a high heterogeneity. Russell et al. showed that clonal populations derived from BM-MSC after sorting for the surface expression of CD146 can be divided into different groups with tripotent (CD146^high^) and bipotent or unipotent (CD146^low^) differentiation potential [33], correlating with the observed difference between CB-MSC and USSC regarding CD146-expression and capacity of adipogenic differentiation. 

To examine a potential heterogeneity in the different populations, 623 clonal populations from 12 different cord blood-derived cell lines were generated by single-cell isolation applying the AVISO CellCelector and analyzed with regard to their differentiation potential, as well as to their proliferation capacity and to their expression profile. 

In addition, new cell lines were generated applying the so-called cloning cylinders to generate cell lines from single colonies. 

The proliferation potential of USSC-derived clonal populations, whether from single cells or colonies, was higher than in CB- or BM-MSC-derived clonal populations, resulting in faster growth and higher total cell numbers achieved before the cells became senescent.

## 6. Differentiation Potential

USSC and CB-MSC, as well as clonal populations, were differentiated *in vitro* according to previously published, protocols, while BM-MSCs were used as control. 

Osteogenic differentiation was performed in a mineralization assay applying ascorbic-acid, beta-glyceroephoshphat, and dexamethason. After 14 days of differentiation, osteogenic specific gene expression was analyzed using quantitative real-time PCR analysis. The osteogenic specific genes *runt-related transcription factor 2* (RUNX2), *osteocalcin* (OC), *bone sialoprotein *(BSP), *osterix* (OSX), and *bone morphogenetic proteins* (BMPs) were already highly expressed in the undifferentiated cell populations. The calcification of the cells after 14 days of differentiation was accompanied by an upregulation of BMP, and OC expression, while the regulation of RUNX2, BSP and OSX was not consistent between the different populations. The verification of the osteogenic specific calcification was performed by Alizarin Red as well as by von Kossa staining. The staining intensity of USSC and CB-MSC was higher than in BM-MSC but varied between the different clonal populations analyzed [17, 31]. 

The chondrogenic differentiation potential was examined applying the pellet culture model as described by Johnstone et al. [34]. The differentiation media, containing *tumour growth factor* (TGF) beta1, ascorbic acid phosphate, sodium pyruvate, and dexamethason, was changed three times a week for a time interval of 21 days. To evaluate the differentiation state, quantitative real-time PCR analysis of *SRY (sex determining region Y)-box 9* (SOX9) expression and Alcian-Blue staining of the chondrogenic specific proteoglycans was performed. No visible differences in the differentiation potential of USSC, CB-MSC, corresponding clones, and BM-MSC were observed [31]. 

The adipogenic differentiation potential of USSC and CB-MSC was assessed by cultivation in medium containing insulin, *isobutylmethylxhanthin* (IBMX), indomethacin and dexamethason for 21 days and subsequent staining of lipid-filled vacuoles by Oil Red O, as well as PCR analysis of the specific genes *peroxisome-proliferator-activated-receptor-gamma 2* (PPAR*γ*2), *fatty acid binding protein *4 (FABP4), *perilipin* (PLIN), and *adiponectin* (ADIPOQ). In accordance with the described classification criteria, USSC and USSC-derived clonal populations showed neither formation of lipid-filled vacuoles nor expression of any adipogenic specific genes, whereas CB-MSC as well as BM-MSC were clearly positive for Oil Red O-staining as well as expression of the corresponding genes. 

## 7. Potential Clinical Use of USSC and CB-MSC

Currently, a wide series of clinical trials with MSC is in progress, for example, treatment of Parkinson, multiple sclerosis, osteoarthritis, diabetes, or spinal cord injury (http://www.clinicaltrials.gov/). Regarding therapeutic appliance, it is mandatory to exclude any potentially harmful property from the applied cells. This includes surveillance of production under clinical conditions, sterility controls, analysis for viral markers and genetic testing (e.g., for a stable karyotype), as well as a complete documentation.

USSC and CB-MSC can be generated under GMP (*good manufacturing practice*)-grade conditions by applying an automated processing protocol. The SEPAX-device, which is also an established system for the processing of CB-transplants, automatically isolates the MNC in a closed system [35]. By using the Cell Stack System, cells can theoretically be expanded up to 10^15^ cells. In initial trials, no differences in the generation frequency or in the quality of the cells in comparison to cell lines generated under standard laboratory conditions were observed [36], which is the first step for cord blood-derived stromal cells to be applicable for clinical treatments.

CB-derived stromal cells have also been analyzed in a series of preclinical models. The osteogenic differentiation potential of USSC and CB-MSC has been proven *in vitro* [15, 17, 31, 37]. To evaluate the potential of ectopic bone formation, CB-derived stromal cells were injected in an immune-deficient rat model on an insoluble bone matrix [38]. The radiological examination revealed a 10 times higher calcification observed in USSC than in ESC treated rats. 

Furthermore, it was demonstrated that USSC can be differentiated into cells with neuronal characteristics [39]. The evidence for *in vivo* neuronal differentiation of CB-derived stromal cells is still lacking confirmation, though transplantation of USSC in an immune-deficient rat with an acute traumatic spinal cord injury led to a migration of these cells to the site of injury and was followed by an enhanced axonal regrowth and reduction of the lesion site [40]. However, no differentiation of the CB cells into neuronal cells could be detected *in vivo*, which leads to the assumption that this might be a paracrine effect. 

Another potential therapeutical appliance of these cells is post myocardial infarction. USSCs possess the ability to differentiate into cells with a cardiac phenotype *in vitro* after coculture with neonatal rat cardiomyocytes and also after injection into a rat model, although the number of differentiated cells was rather low [41]. However, in a porcine acute myocardial infarction model, the transplantation of CB-derived stromal cells led to a reduced scar formation and left-ventricular dilation. This was accompanied by an improved left-ventricular function [42] and subsequently a higher amount of energy reserves at the site of infarction [43] as compared to the control group.

In addition, MSC-like cells can potentially be suitable as supportive means in transplantation of haematopoietic stem cells. As previously described, CB-derived stromal cells (and especially USSCs) possess the ability to support the expansion of CD34^+^ cells in cocultures and lead to improved engraftment of human haematopoietic cells when coinjected into immunodeficient mice. Since the time between transplantation and reconstitution of the patient is most critical, clinical use of USSC might help in reducing mortality due to early infections after transplantation.

Finally, it has also been already demonstrated before that USSC can be differentiated into hepatic like cells, displaying the typical phenotype and a characteristic gene expression profile [44, 45]. *In vivo* this was evidenced in an *in utero* sheep model, where CB-derived stromal cells differentiated into hepatic cells [15]. In adult sheep, USSCs were able to support the hepatic regeneration after portal embolization of the liver; though similar to the effect after cardiac infarction, this seems to be ascribed to a paracrine mechanism [46].

It has to be mentioned, that in general pure paracrine effects without engraftment of the stromal cells might be beneficial and desired, and therefore reducing risks due to unwanted engraftment (e.g., in heart or brain) or other intrinsic properties of the stromal cells.

In summary, these data clearly show that CB-derived stromal cells provide a promising source for a variety of potential clinical applications. 

## 8. UC-MSC 

One remaining question was whether the cell lines generated from cord blood are truly derived from cells circulating in the blood. Critics pointed out that tissue-derived stromal cells might be carried over during the punctuation of the umbilical vein, especially since MSC-like cells isolated from the Wharton's jelly have been described [47–49]. To address this issue, these so-called umbilical cord multipotent stromal cells (UC-MSCs) were generated directly from the extra-embryonic tissue of the umbilical cord according to the protocol as first published by Reinisch and Strunk [50] and then compared to USSC, CB-MSC, and BM-MSC in regard to the morphology, the immunophenotype, the gene expression, and the differentiation potential. 

These cell lines displayed the same fibroblastoid morphology as MSC, but already in the immunophenotype, a first difference was detectable. CD56, described as being expressed on myogenic cells [51], was expressed on the surface of UC-MSC but not on USSC, CB-MSC, or BM-MSC. However, UC-MSCs lack the expression of myogenic-specific markers, such as *myogenic factor 5* (MYF5) and *myosin heavy chain 1* (MYH1). 

Similar to cord blood-derived cell lines, UC-MSC showed a higher proliferation potential than BM-MSC regarding growth rate and expansion before senescence. In contrast to USSC, CB-MSC, and BM-MSC, UC-MSC had neither the ability to differentiate along the adipogenic, nor the chondrogenic or osteogenic lineage *in vitro* as well as *in vivo* (personal communication with P. Bianco, La Sapienza, Rome) and corroborated the data of Kaltz et al. [47].

Although UC-MSCs lacked the multipotent differentiation capacity, they were able to support the proliferation of CD34^+^ cells *in vitro* leading to high expansion rates, but preliminary data suggests that resulting cell populations differ from those obtained on other feeder cells, such as USSC, CB-MSC, or BM-MSC. 

## 9. Hierarchy of USSC and CB-MSC Based on the HOX Expression Profile

In summary, the data reviewed here clearly demonstrate that CB contains at least two different stromal cell populations that can be discriminated by their HOX, DLK-1, and CD146 expression profile and functionally by their adipogenic differentiation potential and the support of haematopoiesis. It has been shown before that CB-derived stromal cells differ from BM-MSC with regard to their transcriptome [52] and their proteome [53]. In 2010, for the first time, a description of different MSC subpopulations in cord blood was published [17, 28]. 

**Figure 1:**
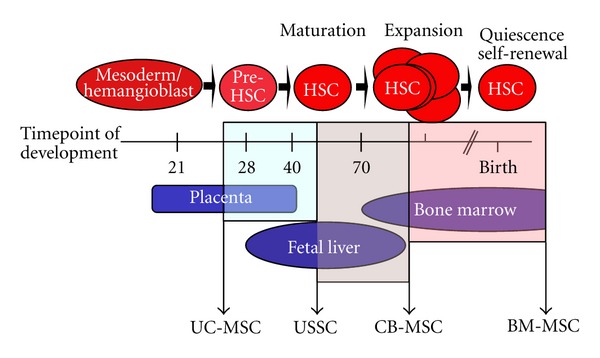
Postulated hierarchy of CB-derived stromal cells (USSC, CB-MSC) in comparison to BM-MSC and stromal cells isolated from the umbilical cord based on specific properties.

With focus on the haematopoiesis during fetal development, the data presented here allow a potential model of a hierarchy as shown in Figure 1.

Together with their high DLK-1 expression and the negative HOX code, as well as their high haematopoiesis-supporting capacity, USSCs have several overlapping features with fetal liver cells. 

CB-MSCs, by contrast, resemble more the adult BM-MSC, as both populations are characterized by a high adipogenic differentiation potential *in vitro* in accordance with the lack of DLK-1 expression, a strong CD146 expression, and a positive expression profile regarding the HOX genes. 

UC-MSC is a completely different cell population with the lack of the so-called MSC differentiation potential. Therefore, these extra-embyronic cells should not be directly compared to MSC-like cells at all.
